# Distributed Two-Dimensional MUSIC for Joint Range and Angle Estimation with Distributed FMCW MIMO Radars

**DOI:** 10.3390/s21227618

**Published:** 2021-11-16

**Authors:** Jiho Seo, Jonghyeok Lee, Jaehyun Park, Hyungju Kim, Sungjin You

**Affiliations:** 1 Division of Smart Robot Convergence and Application Engineering, Department of Electronic Engineering, Pukyong National University, Busan 48513, Korea; sjs9575@naver.com (J.S.); whdgur547@naver.com (J.L.); 2 Communication & Media Research Laboratory, Radio & Satellite Research Division, Electronics and Telecommunications Research Institute, Daejeon 34129, Korea; kimhyungju@etri.re.kr (H.K.); sjyou@etri.re.kr (S.Y.)

**Keywords:** FMCW MIMO radar, distributed radars, joint range and angle estimation, 2D MUSIC algorithm, coordinate transformation

## Abstract

To estimate range and angle information of multiple targets, FMCW MIMO radars have been exploited with 2D MUSIC algorithms. To improve estimation accuracy, received signals from multiple FMCW MIMO radars are collected at the data fusion center and processed coherently, which increases data communication overhead and implementation complexity. To resolve them, we propose the distributed 2D MUSIC algorithm with coordinate transformation, in which 2D MUSIC algorithm is operated with respect to the reference radar’s coordinate at each radar in a distributed way. Rather than forwarding the raw data of received signal to the fusion center, each radar performs 2D MUSIC with its own received signal in the transformed coordinates. Accordingly, the distributed radars do not need to report all their measured signals to the data fusion center, but they forward their local cost function values of 2D MUSIC for the radar image region of interest. The data fusion center can then estimate the range and angle information of targets jointly from the aggregated cost function. By applying the proposed scheme to the experimentally measured data, its performance is verified in the real environment test.

## 1. Introduction

Recently, frequency-modulated continuous waveform (FMCW) radar has been widely exploited in military and automotive radar sensing/imaging systems [1,2,3,4] because it has advantages in the fast-processing time and the robustness to harsh environmental conditions. Accordingly, to estimate the angle with adequate spatial resolution, FMCW multiple-input multiple-output (MIMO) radar has been studied in [5,6,7,8] (and references therein), in which multiple $M_{t}$ transmitters and $M_{r}$ receivers are co-located and the orthogonal waveforms are transmitted from multiple transmit antennas. Then, the reflected signals at the receiver can be equivalently modeled as the received signal from the virtual array with $M_{t}M_{r}$ antenna elements.

To estimate the range and angle information of multiple targets with the FMCW MIMO radar, two-dimensional multiple signal classification (2D MUSIC) algorithms can be exploited [9,10,11,12], in which the covariance matrix of the received signal is estimated for the subspace-based signal processing. We note that the 2D MUSIC algorithm is widely exploited to estimate 2D directions of arrival (DOA) [13,14,15,16], where the elevation and the azimuth angles are jointly estimated by exploiting the planar array antenna. The 2D MUSIC algorithm can also be exploited to estimate the range and angle parameters. In [9], 2D MUSIC algorithm is combined with a FFT-based parameter estimator to reduce the computational complexity of high-resolution 2D MUSIC algorithm. In [10], the weighted 2D root MUSIC algorithm is developed for the joint angle-Doppler estimation. In [11], a reduced-dimension MUSIC algorithm for near-field source localization is proposed, and in [12], a cascade angle estimation algorithm exploiting CAPON and Beam space MUSIC is developed. In addition, to estimate the range and velocity of multiple targets with OFDM radar or to estimate the azimuth and elevation angles of targets, 2D MUSIC algorithm is exploited [17,18]. Specifically, in [17], 2D MUSIC algorithm is exploited for the joint range-velocity estimation, and in [18], 2D MUSIC algorithm is combined with compressive sensing to reduce the system complexity in the joint estimation of azimuth and elevation angles. Furthermore, to improve the estimation accuracy, the received signals from multiple FMCW MIMO radars are collected at the data fusion center and processed coherently [19,20,21], which may increase the data communication overhead and the implementation complexity. In [22], by implementing a low phase noise FMCW radar, the applicability of highly integrated sensors for cooperative bistatic radar networks is demonstrated.

In this paper, to estimate the range and angle information of multiple targets with distributed FMCW MIMO radars, we propose the distributed 2D MUSIC algorithm with coordinate transformation. Specifically, in our proposed scheme, the coordinate at each FMCW MIMO radar is first transformed into the reference radar’s coordinate and then the 2D MUSIC algorithm is separately applied to the received FMCW signal at each distributed radar with respect to the reference radar’s coordinate. Accordingly, rather than reporting the raw data of the received signal to the data fusion center [19,20,21], the distributed radars forward their local cost function values of the 2D MUSIC for the radar image region of interest, resulting in the reduction of data communication burden for the cooperation. Furthermore, because the local cost function values are computed with respect to the same coordinate, their weighted sum can be computed at the data fusion center, and the range and angle information of multiple targets is jointly estimated from the aggregated cost function. Through the computer simulations, the estimation performance of the proposed distributed 2D MUSIC is verified. That is, when the proposed distributed 2D MUSIC algorithm is exploited, high-resolution radar images can be achieved having narrow-width peaks associated with the targets. Accordingly, it can be found that the proposed algorithm shows lower root mean square error (RMSE) than the conventional method especially at low signal-to-noise ratio (SNR). In addition, by applying the proposed scheme to the experimentally measured data, its performance is also verified in the experimentally test.

The rest of this paper is organized as follows. In Section 2, the system model for the distributed FMCW MIMO radar is introduced and the reformulation of received signal is represented to make the signal which is used in the joint range and angle estimation. In Section 3, the conventional 2D MUSIC algorithm is briefly introduced and the distributed 2D MUSIC algorithm with coordinate transformation is proposed to estimate range and angle of multiple targets without sharing all the received signals at the distributed FMCW MIMO radars. In Section 4, we provide several simulation results, and in Section 5, we experimentally demonstrate the proposed scheme with real data measured via two W-band FMCW radars. In Section 6, we give our conclusion.

## 2. System Model for Distributed FMCW MIMO Radar System

**Figure 1:**
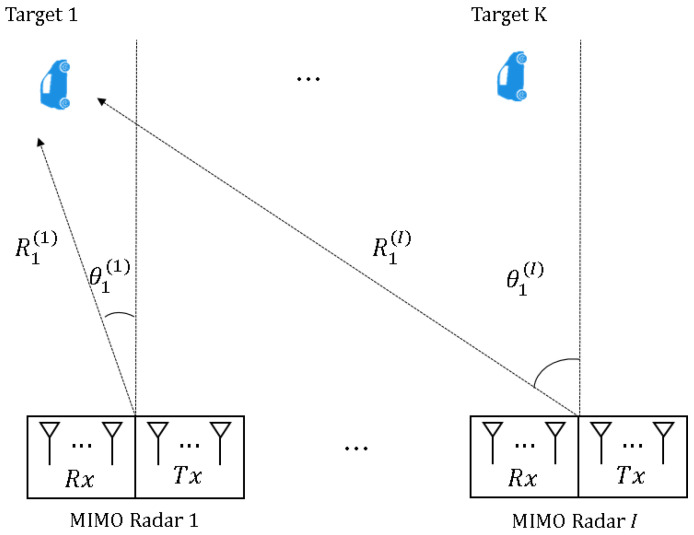
Distributed FMCW MIMO radar and the multiple target environment.

Figure 1 shows the distributed FMCW MIMO radar system with multiple targets in two-dimensional space. Specifically, each FMCW MIMO radar consists of $M_{t}$ number of transmit (Tx) antennas and $M_{r}$ number of receive (Rx) antennas, and *K* number of targets having the angle of $\theta_{k}^{\left(i\right)}$ and the range of $R_{k}^{\left(i\right)}$ with respect to the *i*th FMCW MIMO radar are randomly located with far-field assumption.

### 2.1. Transmitted/Received Signal Model at Distributed FMCW MIMO Radar

We consider that the $m_{t}$th Tx antenna element of the the *i*th radar transmits FMCW signal within a pulse duration $T_{PR}$. Then, the associated FMCW signal can be expressed as

(1) $$s_{m_{t}}^{\left(i\right)}\left(t\right)=\exp\left\{j\left(2\pi \left(f_{c}+\Delta f_{c}\left(m_{t}-1\right)+\Delta f_{d}\left(i-1\right)\right)t+\pi \alpha t^{2}\right)\right\},0\leq t\leq T_{PR},$$

where $f_{c}$ is the carrier frequency and α is the chirp rate. In the distributed FMCW MIMO radar system, frequency offset $\Delta f_{c}$ (i.e., $\Delta f_{d}$) is introduced in (1) to avoid the intra-radar interference caused by the Tx antennas within each FMCW MIMO radar (i.e., the inter-radar interference caused by the Tx antennas from different FMCW MIMO radars). When the transmit signal $s_{m_{t}}^{\left(i\right)}\left(t\right)$ is reflected from the *k*th target, the reflected signal is received at each the $m_{r}$th Rx antenna of the *i*th radar, which is expressed as

(2) $$r_{m_{r}}^{\left(i\right)}\left(t\right)=\sum_{k=1}^{K}\sum_{m_{t}=1}^{M_{t}}\gamma_{k}^{\left(i\right)}s_{m_{t}}^{\left(i\right)}\left(t-\tau_{m_{r}m_{t}k}^{\left(i\right)}\right)+n_{m_{r}}^{\left(i\right)}\left(t\right),$$

where $n_{m_{r}}^{\left(i\right)}\left(t\right)$ denotes the additive white Gaussian noise. Note that ${\gamma_{k}}^{\left(i\right)}$ implies the target reflection coefficient and $\tau_{m_{r}m_{t}k}^{\left(i\right)}$ is the propagation time delay. Here, it is assumed that the antenna gain and the path-loss are also reflected in ${\gamma_{k}}^{\left(i\right)}$ and $\tau_{m_{r}m_{t}k}^{\left(i\right)}$ is the time which the signal from the $m_{t}$th transmit antenna at the *i*th radar was reflected to the *k*th target and received by the $m_{r}$th received antenna. Then, assuming that the relative velocity of the *k*th target with respect to the *i*th target is $v_{k}^{\left(i\right)}$, we can have

(3) $${\gamma_{k}}^{\left(i\right)}=\frac{G}{{\left(R_{m_{t}k}^{\left(i\right)}\right)}^{2}{\left(R_{m_{r}k}^{\left(i\right)}\right)}^{2}}\approx \frac{G}{{\left(R_{0k}^{\left(i\right)}\right)}^{4}}\gamma_{k},$$

and

(4) $$\begin{array}{ccc} \tau_{m_{r}m_{t}k}^{\left(i\right)}\left(t\right) & = & \frac{2}{c}\left(\frac{R_{m_{t}k}^{\left(i\right)}+R_{m_{r}k}^{\left(i\right)}}{2}+\frac{v_{k}^{\left(i\right)}}{2}t\right)\\ & \approx & \frac{2}{c}\left(R_{0k}^{\left(i\right)}+d\left(m-1\right)\sin\theta_{k}^{\left(i\right)}+\frac{v_{k}^{\left(i\right)}}{2}t\right), \end{array}$$

where *G* and $\gamma_{k}$ are the antenna gain and the reflection gain of the *k*th target, respectively. In this paper, ${\left(R_{m_{t}k}^{\left(i\right)}\right)}^{2}{\left(R_{m_{r}k}^{\left(i\right)}\right)}^{2}$ is approximated as ${\left(R_{0k}^{\left(i\right)}\right)}^{4}$ because we assume a far-field distance between the *k*th target and the *i*th radar. Without loss of generality, $\gamma_{k}$ is modeled as a complex Gaussian random variable (i.e., $\gamma_{k}∼\mathrm{CN}\left(0,1\right)$). In (3) and (4), $R_{m_{t}k}^{\left(i\right)}$ (resp., $R_{m_{r}k}^{\left(i\right)}$) is the distance between the $m_{t}$th Tx (resp., the $m_{r}$th Rx) antenna at the *i*th radar and the *k*th target. Here, *d* is the inter-antenna spacing when the virtual antenna array is formed as a linear array by properly locating Tx/Rx antennas [23]. Throughout the paper, is is assumed that d=λ/2, where λ is the wavelength of the FMCW radar waveform. In the last approximation in (4), $R_{0k}^{\left(i\right)}$ is the distance between the position of the reference element (i.e., the first element in the virtual array) in the *i*th radar and the *k*th target. Here, we use the virtual element index $m=1,\cdots ,M_{r}M_{t}$ instead of Tx/Rx indices. Then, (4) can be rewritten as

(5) $$\tau_{mk}^{\left(i\right)}=\tau_{0k}^{\left(i\right)}+\frac{2}{c}\left(d\left(m-1\right)\sin\theta_{k}^{\left(i\right)}\right)+\frac{v_{k}^{\left(i\right)}}{c}t,$$

where $\tau_{0k}^{\left(i\right)}=2R_{0k}^{\left(l\right)}/c$, $m=1,\ldots ,M_{t}M_{r}$.

The received baseband signal at each *i*th radar is then given as

(6) $$\begin{array}{ccc} x_{m}^{\left(i\right)}\left(t\right) & = & LP\{r_{m_{r}}^{(i)*}\left(t\right)s_{m_{t}}^{\left(i\right)}\left(t\right)\}\\ & = & \sum_{k=1}^{K}\sum_{m_{t}=1}^{M_{t}}\gamma_{k}^{\left(i\right)}\exp\{j\left(2\pi \left(f_{c}+\Delta f_{c}\left(m_{t}-1\right)+\Delta f_{d}\left(i-1\right)\right)\tau_{mk}^{\left(i\right)}+\pi \alpha t^{2}\right)\\ & + & 2\pi \alpha \tau_{mk}^{\left(i\right)}t-\pi \alpha {\left(\tau_{mk}^{\left(i\right)}\right)}^{2})\}+n_{m}^{\left(i\right)}\left(t\right)\\ & \approx & \sum_{k=1}^{K}\gamma_{k}^{\left(i\right)}exp\left\{j\left(2\pi f_{c}\tau_{mk}^{\left(i\right)}+2\pi \alpha \tau_{mk}^{\left(i\right)}t-\pi \alpha {\left(\tau_{mk}^{\left(i\right)}\right)}^{2}\right)\right\}+n_{m}^{\left(i\right)}\left(t\right), \end{array}$$

where $n_{m}^{\left(i\right)}\left(t\right)∼\mathrm{CN}\left(0,\sigma_{n}^{2}\right)$ and LP{·} is the low-pass filter output, which passes the beat frequency component of the received signal. Here, the inner product of the Tx waveform and the Rx signal is the de-ramping process. In addition, because $f_{c}$ is $f_{c}\gg \Delta f_{c}\left(resp,\Delta f_{d}\right)$, we can approximate $x_{m}^{\left(i\right)}\left(t\right)$ as the second term in (6).

### 2.2. Reconfiguration to Received Signals into Discrete-Time Signal Matrix

To express (6) as a discrete time domain signal, $x_{m}^{\left(i\right)}\left(t\right)$ can be sampled with the sampling frequency, $f_{s}=1/T_{s}$ which is given as

(7) $$\begin{array}{ccc} x_{m}^{\left(i\right)}\left[n\right] & = & x_{m}^{\left(i\right)}\left(nT_{s}\right)\\ & \approx & \sum_{k=1}^{K}\gamma_{k}^{\left(i\right)}exp\{j(2\pi f_{c}\left(\tau_{0k}^{\left(i\right)}+\frac{2}{c}\left(d\left(m-1\right)\sin\theta_{k}^{\left(i\right)}\right)+\frac{v_{k}^{\left(i\right)}}{c}nT_{s}\right)\\ & + & j2\pi \alpha \left(\tau_{0k}^{\left(i\right)}+\frac{2}{c}\left(d\left(m-1\right)\sin\theta_{k}^{\left(i\right)}\right)+\frac{v_{k}^{\left(i\right)}}{c}nT_{s}\right)nT_{s}\}+n_{m}^{\left(i\right)}\left[n\right], \end{array}$$

where $n_{m}^{\left(i\right)}\left[n\right]≜n_{m}^{\left(i\right)}\left(nT_{s}\right)$. Here, $\tau_{mk}^{\left(i\right)}$ in (5) is substituted into (6) and the second order term $\pi \alpha {\left(\tau_{mk}^{\left(i\right)}\right)}^{2}$ in (6) is ignored. Furthermore, for far-field target range, $\tau_{0k}^{\left(i\right)}\gg \frac{2}{c}\left(d\left(m-1\right)\sin\theta_{k}^{\left(i\right)}+\frac{v_{k}^{\left(i\right)}}{c}nT_{s}\right)$ and therefore $x_{m}^{\left(i\right)}\left[n\right]$ in (7) can be approximated as

(8) $$x_{m}^{\left(i\right)}\left[n\right]\approx \sum_{k=1}^{K}{\bar{\gamma }}_{k}^{\left(i\right)}exp\left\{j2\pi f_{c}\left(\frac{2}{c}\left(d\left(m-1\right)\sin\theta_{k}^{\left(i\right)}\right)+\frac{v_{k}^{\left(i\right)}}{c}nT_{s}\right)+j2\pi \alpha \tau_{0k}^{\left(i\right)}nT_{s}\right\},$$

where ${\bar{\gamma }}_{k}^{\left(i\right)}=\gamma_{k}^{\left(i\right)}exp\left\{j2\pi f_{c}\tau_{0k}^{\left(i\right)}\right\}$. In this paper, total *S* FMCW pulses are collected at the receiver and by introducing the pulse index *s*, the discrete signal $x_{m}^{\left(i\right)}\left[n,s\right]$, the *n*th sample of the *s*th FMCW pulses, can then be represented as

$$\begin{array}{ccc} x_{m}^{\left(i\right)}\left[n,s\right] & \approx & x_{m}^{\left(i\right)}\left(nT_{s}\right){|}_{s_{th}pulse}\\ & \approx & \sum_{k=1}^{K}{\bar{\gamma }}_{k}^{\left(i\right)}exp\{j2\pi f_{c}\left(\frac{2}{c}\left(d\left(m-1\right)\sin\theta_{k}^{\left(i\right)}+\frac{v_{k}^{\left(i\right)}}{c}\left(sT_{PR}+nT_{s}\right)\right)+j2\pi \alpha \tau_{0k}^{\left(i\right)}nT_{s}\right\}\\ & = & \sum_{k=1}^{K}{\bar{\gamma }}_{k}^{\left(i\right)}exp\{j2\pi f_{c}\left(\frac{2}{c}\left(d\left(m-1\right)\sin\theta_{k}^{\left(i\right)}+\frac{v_{k}^{\left(i\right)}}{c}\left(sT_{PR}\right)\right)+j2\pi \left(\alpha \tau_{0k}^{\left(i\right)}+\frac{v_{k}^{\left(i\right)}}{\lambda }\right)nT_{s}\right\}. \end{array}$$

Then, from $exp\{j2\pi f_{c}(2/c\left(d\left(m-1\right)\sin\theta_{k}^{\left(i\right)}\right\}$ in (9), by introducing the array response vector $a(\theta_{k}^{\left(i\right)})$, $x_{m}^{\left(i\right)}\left[n,s\right]$ can be rewritten as a vector form. Specifically, for a linear array antenna, the array steering vector is given as

(9) $$a\left(\theta_{k}^{\left(i\right)}\right)=\left[1,exp\left\{2\pi \frac{2d}{\lambda }\right)\sin\theta_{k}^{\left(i\right)}\right\},\ldots ,exp\left\{2\pi \frac{2d(M-1)}{\lambda }\right)\sin\theta_{k}^{\left(i\right)}{\}]}^{T},$$

and $x_{m}^{\left(i\right)}\left[n,s\right]$ can then be vectorized as

(10) $$\begin{array}{ccc} x^{\left(i\right)}\left[n,s\right] & = & \left[\begin{array}{c} x_{1}^{\left(i\right)}\left[n,s\right]\\ \vdots \\ x_{M}^{\left(i\right)}\left[n,s\right] \end{array}\right]\\ & = & \sum_{k=1}^{K}{\bar{\gamma }}_{k}^{\left(i\right)}a\left(\theta_{k}^{\left(i\right)}\right)exp\left\{j2\pi \frac{v_{k}^{\left(i\right)}}{\lambda }sT_{PR}+j2\pi \left(\alpha \tau_{0k}^{\left(i\right)}+\frac{v_{k}^{\left(i\right)}}{\lambda }\right)nT_{s}\right)\}, \end{array}$$

**Figure 2:**
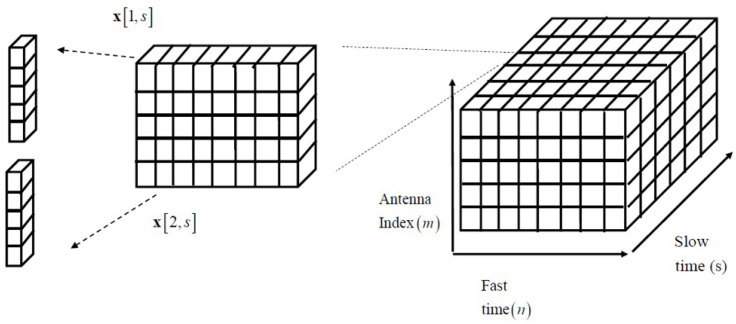
Pictorial description for $x^{\left(i\right)}\left[n,s\right]$ and ${\tilde{x}}^{\left(i\right)}\left[s\right]$.

As shown in Figure 2, by stacking $x^{\left(i\right)}\left[n,s\right]$ for n=1,…,N, we can further have

(11) $$\begin{array}{ccc} {\bar{x}}^{\left(i\right)}\left[s\right] & = & \left[\begin{array}{c} x^{\left(i\right)}\left[1,s\right]\\ \vdots \\ x^{\left(i\right)}\left[N,s\right] \end{array}\right]\\ & = & \sum_{k=1}^{K}{\bar{\gamma }}_{k}^{\left(i\right)}b\left(\tau_{0k}^{\left(i\right)},v_{k}^{\left(i\right)}\right)⊗a\left(\theta_{k}^{\left(i\right)}\right)exp\left\{j2\pi \frac{v_{k}^{\left(i\right)}}{\lambda }sT_{PR}\right\}, \end{array}$$

where the notation ⊗ is Kronecker product operator and for $\alpha \tau_{0k}^{\left(i\right)}\gg v_{k}^{\left(i\right)}/\lambda$, $b(\tau_{0k}^{\left(i\right)},v_{k}^{\left(i\right)})$ can be approximated as

(12) $$\begin{array}{ccc} b(\tau_{0k}^{\left(i\right)},v_{k}^{\left(i\right)}) & = & C[1\cdots \exp{\left\{j2\pi \left(\alpha \tau_{0k}^{\left(i\right)}+\frac{v_{k}^{\left(i\right)}}{\lambda }\right)\left(N-1\right)T_{s}\right]}^{T}\\ & \approx & C[1\cdots \exp{\left\{j2\pi \left(\alpha \tau_{0k}^{\left(i\right)}\right)\left(N-1\right)T_{s}\right]}^{T}\left(≜b\left(\tau_{0k}^{\left(i\right)}\right)\right), \end{array}$$

where $C≜\exp\left\{j2\pi \left(\alpha \tau_{0k}^{\left(i\right)}+v_{k}^{\left(i\right)}/\lambda \right)T_{s}\right\}\approx \exp\left\{j2\pi \left(\alpha \tau_{0k}^{\left(i\right)}\right)T_{s}\right\}$. We note that $b(\tau_{0k}^{\left(i\right)})$ is the fast-time array response vector associated with $\tau_{0k}^{\left(i\right)}$, while $a(\theta_{k}^{\left(i\right)})$ is a spatial response vector associated with $\theta_{k}^{\left(i\right)}$. In Figure 2, the pictorial description for ${\bar{x}}^{\left(i\right)}\left[s\right]$ and $x^{\left(i\right)}\left[n,s\right]$ in (10) and (11). Finally, by concatenating ${\bar{x}}^{\left(i\right)}\left[s\right]$ for s=1,⋯,S, we have a discrete time de-ramped signals as

(13) $$X^{\left(i\right)}=\left[{\bar{x}}^{\left(i\right)}\left[1\right],\cdots ,{\bar{x}}^{\left(i\right)}\left[S\right]\right]$$

## 3. Distributed 2D MUSIC Algorithm for Joint Range and Angle Estimation

### 3.1. 2D MUSIC Algorithm with a Single FMCW MIMO Radar

In the FMCW MIMO radar system, ranges and azimuth angles of multiple targets are jointly estimated through the 2D FFT operation, but to obtain high-resolution estimates of the ranges and azimuth angles of multiple targets, we develop the subspace-based 2D MUSIC algorithm. We also note that, in [24], 2D MUSIC algorithm is exploited to estimate the angles and Doppler frequencies of the targets. First, the sample covariance matrix of $X^{\left(i\right)}$ is computed from (13) and its eigenvalue decomposition (EVD) can be given as

(14) $${\bar{R}}^{\left(i\right)}=\frac{1}{S}X^{\left(i\right)}X^{(i)H}=\left[\begin{array}{cc} E_{s}^{\left(i\right)} & E_{n}^{\left(i\right)} \end{array}\right]\left[\begin{array}{cc} \Lambda_{s}^{\left(i\right)} & 0\\ 0 & \Lambda_{n}^{\left(i\right)} \end{array}\right]{\left[\begin{array}{cc} E_{s}^{\left(i\right)} & E_{n}^{\left(i\right)} \end{array}\right]}^{H},$$

where $E_{s}^{\left(i\right)}\in C^{MN\times K}$ is the matrix whose columns consist of the eigenvectors that span the signal subspace of ${\bar{R}}^{\left(i\right)}$. In addition, the columns $E_{n}^{\left(i\right)}\in C^{MN\times (MN-K)}$ are the eigenvectors that span the noise subspace of ${\bar{R}}^{\left(i\right)}$. Here, $\Lambda_{s}^{\left(i\right)}\in C^{K\times K}$ and $\Lambda_{n}^{\left(i\right)}\in C^{\left(MN-K)\times (MN-K\right)}$ are the diagonal matrices whose diagonal elements consist of the (K,MN−K) eigenvalues of ${\bar{R}}^{\left(i\right)}$. Note that the columns of $E_{n}^{\left(i\right)}$ are orthogonal to those of $E_{s}^{\left(i\right)}$ (equivalently, the signal subspace spanned by the columns of $X^{\left(i\right)}$ in (13)). Accordingly, from (11) and (12), we define f(τ,θ) as

(15) f(τ,θ)≜b(τ)⊗a(θ).

Then, by letting the cost function $J^{\left(i\right)}\left(\tau ,\theta \right)$ as

(16) $$J^{\left(i\right)}\left(\tau ,\theta \right)≜\frac{1}{f^{H}\left(\tau ,\theta \right)E_{n}^{\left(i\right)}E_{n}^{(i)H}f\left(\tau ,\theta \right)},$$

the ranges (more specifically, the time delays associated with the ranges of targets) and angles can be jointly estimated at the *i*th radar as

(17) $$\left({\hat{\tau }}_{k}^{\left(i\right)},{\hat{\theta }}_{k}^{\left(i\right)}\right)=\underset{\tau ,\theta }{\arg\max}J^{\left(i\right)}\left(\tau ,\theta \right),\;\mathrm{for}\;k=1,\ldots ,K,$$

where *K* is the number of targets. When *K* is not known at radar, to estimate the number of signal sources, the minimum description length (MDL) [25,26] can be exploited as

(18) $${\hat{K}}^{\left(i\right)}=\underset{k\in \{1,\ldots ,MN\}}{\arg\min}MDL^{\left(i\right)}\left(k\right),$$

where

(19) $$\begin{array}{ccc} MDL^{\left(i\right)}\left(k\right) & = & -\log{\left(\frac{\prod_{p=k+1}^{MN}{\left(\lambda_{p}^{\left(i\right)}\right)}^{1/(MN-k)}}{\frac{1}{\left(MN-k\right)}\sum_{p=k+1}^{MN}\lambda_{p}^{\left(i\right)}}\right)}^{(MN-k)S}+\frac{1}{2}k\left(2MN-k\right)\log S. \end{array}$$

Here, $\lambda_{p}^{\left(i\right)}$ is the *p*th eigenvalue of ${\bar{R}}^{\left(i\right)}$ in (14).

### 3.2. Distributed 2D MUSIC Algorithm with Coordinate Transformation

To improve the estimation accuracy, the received signals from the distributed FMCW MIMO radars are collected at the data fusion center and processed non-coherently, which requires large data communication overhead and computational complexity burden at the data fusion center. Instead, we propose a distributed 2D MUSIC using coordinate transformation. Note that the range and angle pairs $\left(R_{k}^{\left(i\right)},\theta_{k}^{\left(i\right)}\right)$, k=1,…,K are the coordinates with respect to the *i*th FMCW MIMO radar. Accordingly, in the proposed scheme, the coordinates are transformed with respect to the reference radar before the 2D MUSIC algorithm is processed. That is, rather than forwarding the raw data of the received signal to the data fusion center, each radar performs the 2D MUSIC algorithm with its own received signal using the transformed coordinates.

**Figure 3:**
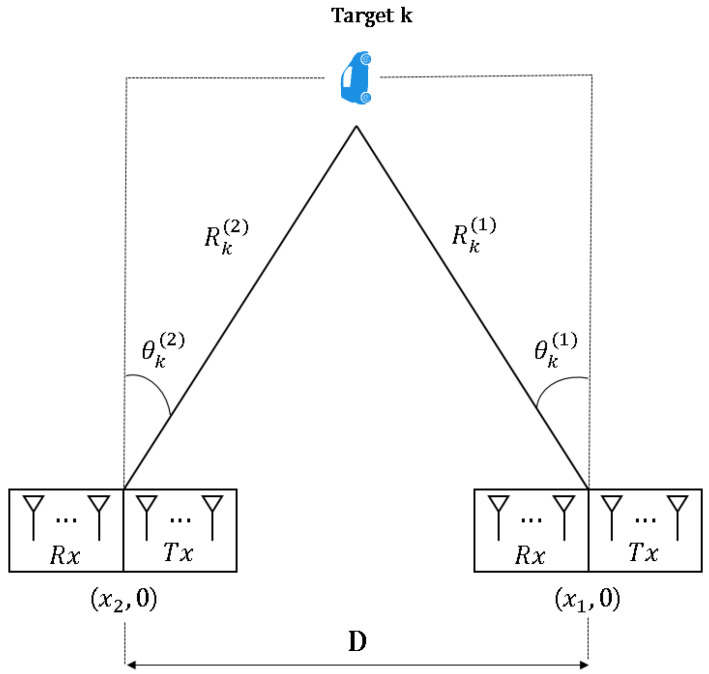
Coordinate transformation in distributed FMCW MIMO radar system.

We consider that the distributed radars are in a straight line (see Figure 3), but it can be easily extended to the cases with their arbitrary locations. Specifically, the *i*th radar is located in $\left(x_{i},0\right)$. Without loss of generality, the radar located at $\left(x_{1},0\right)$ is denoted as the reference radar. From Figure 3, we can have two equations representing the relationship between $\left(R_{k}^{\left(1\right)},\theta_{k}^{\left(1\right)}\right)$ and $\left(R_{k}^{\left(i\right)},\theta_{k}^{\left(i\right)}\right)$ as

(20) $$R_{k}^{\left(i\right)}\cos\theta_{k}^{\left(i\right)}=R_{k}^{\left(1\right)}\cos\theta_{k}^{\left(1\right)}$$

(21) $$R_{k}^{\left(i\right)}\sin\theta_{k}^{\left(i\right)}-R_{k}^{\left(1\right)}\sin\theta_{k}^{\left(1\right)}=D_{i1},$$

where $D_{i1}$ is the distance between the reference radar and the *i*th radar (i.e., $D_{i1}=\left|x_{i}-x_{1}\right|$). Accordingly, $\left(R_{k}^{\left(i\right)},\theta_{k}^{\left(i\right)}\right)$ can be expressed in terms of $\left(R_{k}^{\left(1\right)},\theta_{k}^{\left(1\right)}\right)$ as

(22) $$R_{k}^{\left(i\right)}=\sqrt{{\left(R_{k}^{\left(1\right)}\right)}^{2}+D_{i1}^{2}+2D_{i1}R_{k}^{\left(1\right)}\sin\theta_{k}^{\left(1\right)}}$$

(23) $$\theta_{k}^{\left(i\right)}=\sin^{-1}\frac{\left(x_{1}-x_{i}\right)+R_{k}^{\left(1\right)}\sin\theta_{k}^{\left(1\right)}}{\sqrt{{\left(R_{k}^{\left(1\right)}\right)}^{2}+D_{i1}^{2}+2D_{i1}R_{k}^{\left(1\right)}\sin\theta_{k}^{\left(1\right)}}},$$

Because $\tau_{k}^{\left(i\right)}=2R_{k}^{\left(i\right)}/c$, from (22) and (23), we can express (τ,θ) in the *i*th radar’s coordinate as

(24) $$\begin{array}{c} \tau =\sqrt{{\left(\bar{\tau }c/2\right)}^{2}+D_{i1}^{2}+2D_{i1}\bar{\tau }c/2\sin\theta_{k}^{\left(1\right)}}≜f_{tr}\left(\bar{\tau },\bar{\theta }\right) \end{array}$$

(25) $$\begin{array}{c} \theta =\sin^{-1}\frac{\left(x_{1}-x_{i}\right)+\bar{\tau }c/2\sin\theta_{k}^{\left(1\right)}}{\sqrt{{\left(\bar{\tau }c/2\right)}^{2}+D_{i1}^{2}+2D_{i1}\bar{\tau }c/2\sin\theta_{k}^{\left(1\right)}}}≜g_{tr}\left(\bar{\tau },\bar{\theta }\right), \end{array}$$

where $\left(\bar{\tau },\bar{\theta }\right)$ is based on the reference radar’s coordinate. Then, by substituting (τ,θ) in (24) and (25) into (15) and (16), at the *i*th FMCW MIMO radar, the cost function in (16) is reformulated with respect to the reference radar’s coordinate $\left(\bar{\tau },\bar{\theta }\right)$ as

(26) $${\bar{J}}^{\left(i\right)}\left(\bar{\tau },\bar{\theta }\right)={\bar{J}}^{\left(i\right)}\left(f_{tr}\left(\bar{\tau },\bar{\theta }\right),g_{tr}\left(\bar{\tau },\bar{\theta }\right)\right)=\frac{1}{f^{H}\left(f_{tr}\left(\bar{\tau },\bar{\theta }\right),g_{tr}\left(\bar{\tau },\bar{\theta }\right)\right)E_{n}^{\left(i\right)}E_{n}^{(i)H}f\left(f_{tr}\left(\bar{\tau },\bar{\theta }\right),g_{tr}\left(\bar{\tau },\bar{\theta }\right)\right)}$$

Then, the distributed radars report $J^{\left(i\right)}\left(\bar{\tau },\bar{\theta }\right)$ for the radar image region of interest to the data fusion center. At the data fusion center, the aggregated cost function can be formulated as

(27) $$\bar{J}\left(\bar{\tau },\bar{\theta }\right)=\sum_{i=1}^{L}w^{\left(i\right)}{\bar{J}}^{\left(i\right)}\left(\bar{\tau },\bar{\theta }\right),$$

where $w^{\left(i\right)}$ is the weight for the *i*th local cost function value and it can be determined as being proportional to the received SNR at the *i*th radar,

(28) $$w^{\left(i\right)}=\frac{SNR^{\left(i\right)}}{\sum_{i=1}^{I}SNR^{\left(i\right)}}$$

The delays and angles can then be jointly estimated at the data fusion center as

(29) $$\left({\hat{\bar{\tau }}}_{k},{\hat{\bar{\theta }}}_{k}\right)=\underset{\bar{\tau },\bar{\theta }}{\arg\max}\bar{J}\left(\bar{\tau },\bar{\theta }\right),\;\mathrm{for}\;k=1,\ldots ,K.$$

Note that the distributed radars do not need to report all their measured signals to the data fusion center, but they forward their local cost function (26) for the radar image region of interest. Based on the above description, the proposed distributed 2D MUSIC algorithm with coordinate transformation is summarized in Algorithm 1.
**Algorithm 1** Distributed 2D MUSIC algorithm with coordinate transformation1:The *i*th radar, i=1,⋯,L
2:   Compute ${\bar{R}}^{\left(i\right)}$3:    Estimate the number of targets(${\hat{K}}^{\left(i\right)}$) using MDL criterion in (18).4:   Compute the EVD of ${\bar{R}}^{\left(i\right)}$ as ${\bar{R}}^{\left(i\right)}=E^{\left(i\right)}\Sigma^{\left(i\right)}{E^{\left(i\right)}}^{H}$.5:   Formulate $f_{tr}\left(\bar{\tau },\bar{\theta }\right)$ and $g_{tr}\left(\bar{\tau },\bar{\theta }\right)$ through the coordinate transformation as (24) and (25).6:   Compute the cost function ${\bar{J}}^{\left(i\right)}\left(\bar{\tau },\bar{\theta }\right)$ using $f_{tr}\left(\bar{\tau },\bar{\theta }\right)$ and $g_{tr}\left(\bar{\tau },\bar{\theta }\right)$ as (26).7:   Forward its local cost ${\bar{J}}^{\left(i\right)}\left(\bar{\tau },\bar{\theta }\right)$ for the radar image region of interest.8:The data fusion center:9:   Compute the aggregated cost function as (27)10:   Estimate the parameters $\left({\hat{\bar{\tau }}}_{k},{\hat{\bar{\theta }}}_{k}\right)$ associate with the largest $\hat{K}$ peaks using aggregated cost function as in (29). Here, $\hat{K}=\max_{i=1,\ldots ,L}{\hat{K}}^{\left(i\right)}$.

### 3.3. Discussion

To see the effectiveness of the proposed 2D MUSIC algorithm with coordinate transformation, we look into the application of 2D MUSIC algorithm with the received signal sharing. That is, when the received signals from the distributed radars are transferred perfectly to the data fusion center and the received signals are uncorrelated, we can have the sample covariance matrix of $X={\left[{\left(X^{\left(1\right)}\right)}^{H},\ldots ,{\left(X^{\left(L\right)}\right)}^{H}\right]}^{H}$ as

(30) $$\begin{array}{c} \bar{R}=\frac{1}{S}XX^{H}\approx diag\left\{{\bar{R}}^{\left(1\right)},\ldots ,{\bar{R}}^{\left(L\right)}\right\}. \end{array}$$

and therefore, the eigenvectors that span the noise subspace of $\bar{R}$ can be given as

(31) $$\begin{array}{c} E_{n}=\left[\begin{array}{cccc} E_{n}^{\left(1\right)} & 0 & \cdots & 0\\ 0 & E_{n}^{\left(2\right)} & \cdots & \vdots \\ \vdots & & \ddots & 0\\ 0 & \cdots & 0 & E_{n}^{\left(L\right)} \end{array}\right]. \end{array}$$

Let us define the time and spatial array response vector as

(32) $$\begin{array}{c} f_{T}\left(\bar{\tau },\bar{\theta }\right)={\left[f_{1}^{T}\left(\bar{\tau },\bar{\theta }\right),\ldots ,f_{L}^{T}\left(\bar{\tau },\bar{\theta }\right)\right]}^{T}, \end{array}$$

where $\bar{\tau }$ and $\bar{\theta }$ are, respectively, the delay and the azimuth angle based on the reference radar’s coordinate and $f_{i}\left(\bar{\tau },\bar{\theta }\right)$ is the *i*th relative time and spatial array response vector, where the relative position of the *i*th radar with respect to the reference radar is reflected. We note that $f_{i}\left(\bar{\tau },\bar{\theta }\right)=K_{i}b\left(\tau^{\left(i\right)}\right)⊗a\left(\theta^{\left(i\right)}\right)$ with a complex valued constant $K_{i}$ such that $|K_{i}|=1$. Here, $\tau^{\left(i\right)}$ and $\theta^{\left(i\right)}$ are, respectively, the delay and the azimuth angle based on the *i*th radar’s coordinate. The cost function for the 2D MUSIC with the received signal sharing can then be given as

(33) $$\begin{array}{ccc} {\bar{J}}_{T}\left(\bar{\tau },\bar{\theta }\right) & = & \frac{1}{f_{T}^{H}\left(\bar{\tau },\bar{\theta }\right)E_{n}E_{n}^{H}f\left(\bar{\tau },\bar{\theta }\right)}=\frac{1}{\sum_{i=1}^{L}f^{H}\left(\tau^{\left(i\right)},\theta^{\left(i\right)}\right)E_{n}^{\left(i\right)}E_{n}^{(i)H}f\left(\tau^{\left(i\right)},\theta^{\left(i\right)}\right)}\\ & = & \frac{1}{\sum_{i=1}^{L}f^{H}\left(f_{tr}\left(\bar{\tau },\bar{\theta }\right),g_{tr}\left(\bar{\tau },\bar{\theta }\right)\right)E_{n}^{\left(i\right)}E_{n}^{(i)H}f\left(f_{tr}\left(\bar{\tau },\bar{\theta }\right),g_{tr}\left(\bar{\tau },\bar{\theta }\right)\right)}, \end{array}$$

and accordingly, the delays and angles can be estimated at the data fusion center as

(34) $$\left({\hat{\bar{\tau }}}_{k},{\hat{\bar{\theta }}}_{k}\right)=\underset{\bar{\tau },\bar{\theta }}{\arg\max}J_{T}\left(\bar{\tau },\bar{\theta }\right),\;\mathrm{for}\;k=1,\ldots ,K.$$

We note that (33) is comparable with (27). That is, both (27) and (33) are maximized when $f^{H}\left(f_{tr}\left(\bar{\tau },\bar{\theta }\right),g_{tr}\left(\bar{\tau },\bar{\theta }\right)\right)E_{n}^{\left(i\right)}E_{n}^{(i)H}f\left(f_{tr}\left(\bar{\tau },\bar{\theta }\right),g_{tr}\left(\bar{\tau },\bar{\theta }\right)\right)$ for i=1,…,L simultaneously become close to zero. From the above observation, our proposed 2D MUSIC, which does not require the transfer of the raw data of the received signals to the data fusion center, gives a similar estimation performance compared to the 2D MUSIC with the received signal sharing.

## 4. Simulation Results

To verify the proposed distributed 2D MUSIC algorithm, the computer simulations are performed. Throughout the simulations, we use 77 GHz as the center frequency, 300 MHz as the operating bandwidth, 3.33 μs as the pulse duration, and 600 MHz as the sampling frequency. The number of pulses is set as 100. In addition, the number of time samples is set as 2000 samples per pulse and FMCW MIMO radar is exploited with $M_{t}=2$ and $M_{r}=4$, where the transceiver antennas are placed such that virtual uniform linear antenna array is formed with inter-antenna spacing λ/2, where λ is a wavelength of the FMCW waveform. Throughout the simulations, we assume that Line-of-Sight (LoS) component is retained for each target and there is no multi-path. For the LoS, a typical path-loss exponent is set as 2, but it can be extended to general types of channel model.

### 4.1. The 2D MUSIC-Based Radar Image Comparison

**Figure 4:**
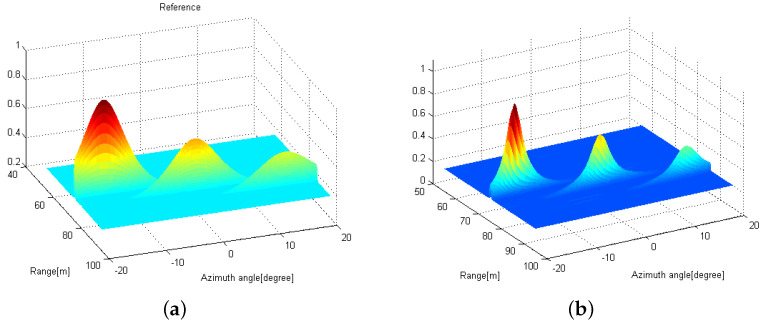
Radar images obtained by using (**a**) the conventional 2D MUSIC with SNR=10 dB and (**b**) the proposed distributed 2D MUSIC with D=5 m and SNR=10 dB.

We consider that three targets are located ($R_{k}$, $\theta_{k}$) = $\left\{\left(75\;m,-15^{\circ }\right),\left(80\;m,0^{\circ }\right),\left(85\;m,15^{\circ }\right)\right\}$ and two distributed FMCW MIMO radars are exploited, where the reference radar is located at the origin point (0,0) m and the other radar is at (D,0) m. Figure 4a shows the radar image at the reference radar based on the conventional 2D MUSIC algorithm in Section 3.1 when the received SNR is 10 dB. Note that three peaks are observed at $\left\{\left(75\;m,-15^{\circ }\right),\left(80\;m,0^{\circ }\right),\left(85\;m,15^{\circ }\right)\right\}$. Interestingly, all peaks have the relatively wide width along the azimuth angle axis, compared to the range axis. This is because the number of elements in the virtual array is much smaller than that of fast-time samples, resulting in the relatively low angle resolution. In Figure 4b, the radar image using the proposed distributed 2D MUSIC with coordinate transformation is shown with D=5 m, when the received SNR is 10 dB at both FMCW MIMO radars. That is, the image is plotted based on (27). We can also find three peaks at $\left\{\left(75\;m,-15^{\circ }\right),\left(80\;m,0^{\circ }\right),\left(85\;m,15^{\circ }\right)\right\}$. Note that the peaks are sharper than those in Figure 4a, which implies that the proposed distributed 2D MUSIC can have a higher angle resolution and lower estimation errors compared to the conventional 2D MUSIC without the received signal sharing.

**Figure 5:**
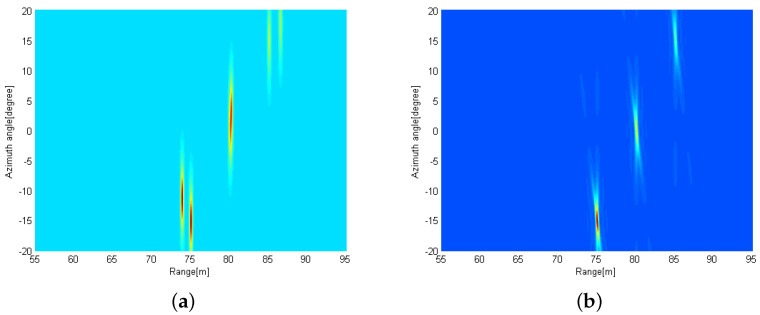
Radar images obtained by using (**a**) the conventional 2D MUSIC and (**b**) the proposed distributed 2D MUSIC in a top view.

In Figure 5, the radar images are shown when (a) the images obtained from two distributed radars are simply averaged and (b) the proposed 2D MUSIC with coordinate transformation is applied using (27). Here, we also consider that three targets are located at ($R_{k}$, $\theta_{k}$) = $\left\{\left(75\;m,-15^{\circ }\right),\left(80\;m,0^{\circ }\right),\left(85\;m,15^{\circ }\right)\right\}$. From Figure 5a, some ghost targets are observed because the coordinates of the distributed radars are not properly aligned. However, in the Figure 5b, three peaks can be found associated with the original target positions with a high resolution.

**Figure 6:**
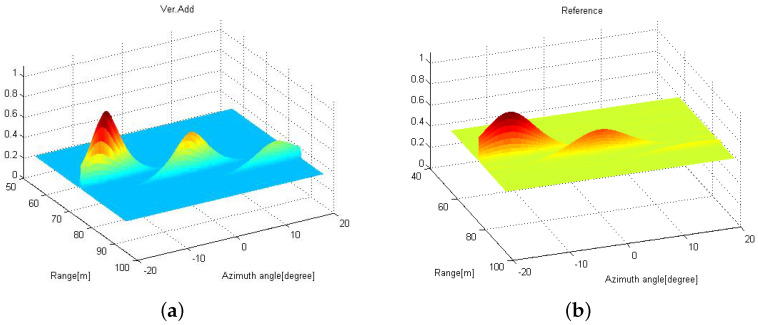
Radar images obtained by using (**a**) the proposed 2D MUSIC and (**b**) the conventional 2D MUSIC when SNR={10,6.6} dB at two different radars.

In Figure 6a, the radar image using the proposed 2D MUSIC algorithm with coordinate transformation is shown with D=1 m, when the received SNRs are different at the distributed radars. Specifically, the received SNR at the reference radar is 6.6 dB, while 10 dB at the other radar. We can also find three peaks at the same point. For comparison purpose, in Figure 6b, the radar image at the reference radar using the conventional 2D MUSIC algorithm is shown. We note that the peaks associated with the targets are not apparent compared to that in Figure 6a.

**Figure 7:**
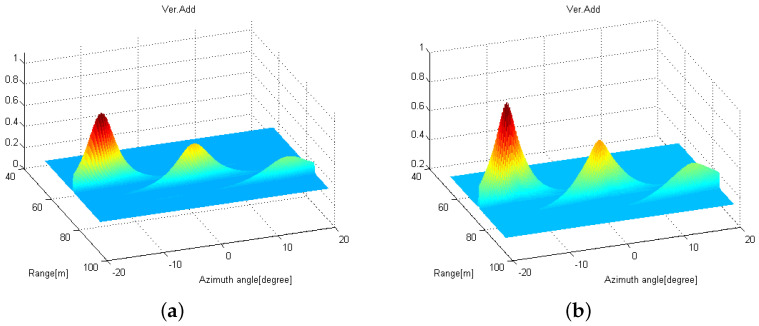
Radar images obtained by using the proposed distributed 2D MUSIC algorithm with (**a**) D=5 m and (**b**) D=10 m.

In Figure 7, the radar images using the proposed distributed 2D MUSIC with coordinate transformation are shown with the different inter-radar spaces (i.e., D={5,10}m). We can also find as the inter-radar space increases, the radar image resolution can be improved.

### 4.2. Mean Square Error Comparison

In this section, the Monte Carlo simulations are carried out to present the performance of the proposed algorithm. As performance measures, we evaluate the RMSEs of range and angle estimation, given as follows:

(35) $$\begin{array}{cc} & RMSE_{r}=\sqrt{\left[\begin{array}{c} \frac{1}{K}\sum_{k=1}^{K}{\left({\hat{\bar{r}}}_{k}-r_{k}\right)}^{2} \end{array}\right]}\\ & RMSE_{\theta }=\sqrt{\left[\begin{array}{c} \frac{1}{K}\sum_{k=1}^{K}{\left({\hat{\bar{\theta }}}_{k}-\theta_{k}\right)}^{2} \end{array}\right]} \end{array}$$

**Figure 8:**
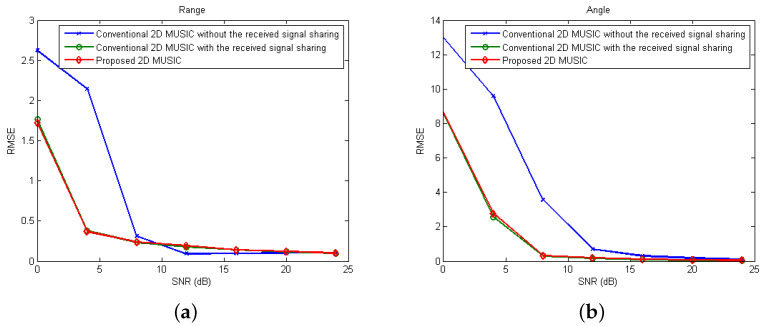
RMSE values of (**a**) range estimation and (**b**) angle estimation over various SNRs.

In Figure 8, we set $M_{t}=2$, $M_{r}=4$, L=2, and K=2 with ($R_{k}$, $\theta_{k}$) = $\left\{\left(70\;m,-15^{\circ }\right),\left(75\;m,15^{\circ }\right)\right\}$. Figure 8a shows the RMSEs of range estimation for various SNRs when two distributed FMCW MIMO radars with D=10 m are exploited with the proposed 2D MUSIC algorithm. For comparison purposes, we also evaluate the RMSEs when the conventional 2D MUSIC with/without the received signal sharing are exploited. From Figure 8a, as SNR increases, the RMSEs for the proposed algorithm and the conventional algorithms with/without received signal sharing decrease, but at low SNR values, the proposed algorithm shows lower RMSEs than the conventional algorithm without received signal sharing. Interestingly, it can be found that the proposed 2D MUSIC algorithm exhibits a similar RMSE performance compared to the 2D MUSIC with the received signal sharing, which coincides with the discussion in Section 3.3. From Figure 8b, RMSEs of angle estimation for the proposed algorithm are lower than those for the conventional algorithm without data sharing, which is a similar observation as that found in Figure 8a.

**Figure 9:**
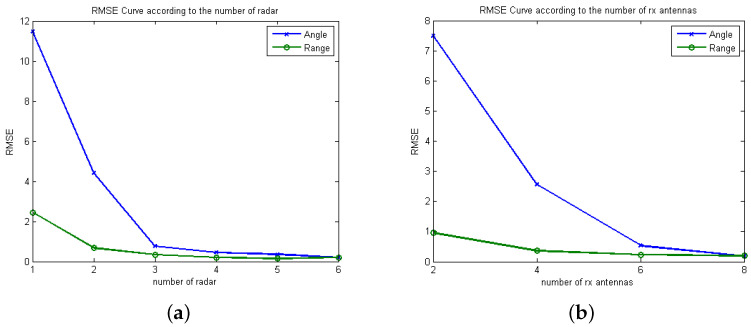
RMSE values of range and angle estimation over various numbers of (**a**) FMCW MIMO radars and (**b**) Rx antennas.

Figure 9 shows the RMSEs of angle and range estimation for various numbers of FMCW MIMO radars and Rx antennas. Specifically, in Figure 9a, the RMSEs for various numbers of FMCW MIMO radars are evaluated when the proposed algorithm is exploited with SNR=4 dB, $M_{t}=2$, $M_{r}=4$. That is, the *i*th radars are located at the point ((i−1)D,0) m with D=1 m. From Figure 9a, as the number of radars increases, RMSEs of both range and angle are decrease. Accordingly, as the number of radars increases, high-resolution images can be achieved having narrow-width peaks associated with the targets, which coincides with the observation in Section 4.1. In Figure 9b, the RMSEs for various numbers of Rx antennas with two FMCW MIMO radars are evaluated. Here, SNR=4 dB, $M_{t}=2$, and D=10 m. Furthermore, from Figure 9b, as the number of Rx antennas increases, RMSEs of both range and angle are decreases.

## 5. Experiment Results

**Figure 10:**
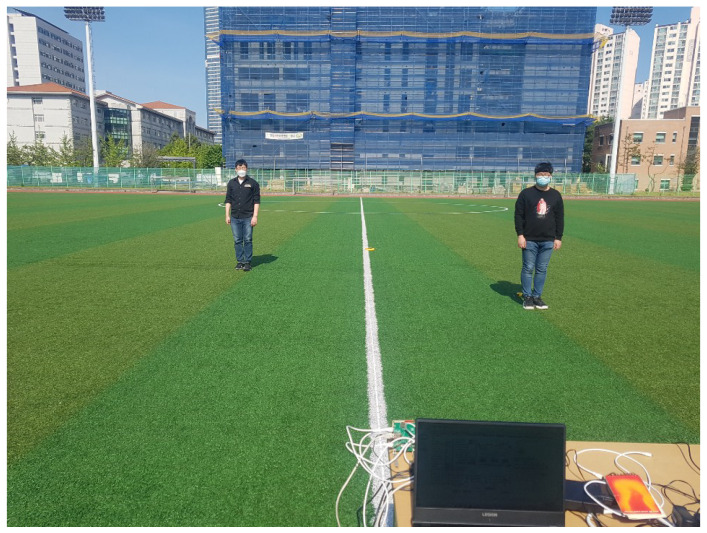
Experiment setup to measure the distributed FMCW radar signals.

In order to experimentally demonstrate the proposed 2D MUSIC algorithm, a distributed FMCW MIMO radar system is set as in Figure 10 by using two W-band FMCW radars (TI AWR-1642) with two Tx and four Rx antennas. Here, two radars are located at (0,0) m and (2,0) m. We consider that two targets are located at $\left(7.76\;m,-14.93^{\circ }\right)$ and $\left(5.39\;m,21.80^{\circ }\right)$ with respect to the reference radar at (0,0) m. The FMCW chirp configuration used in our experiment is shown in Table 1.

**Figure 11:**
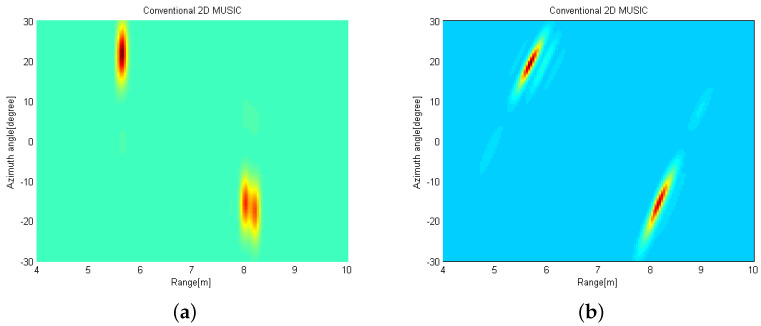
Radar images (**a**) at reference radar by using the conventional 2D MUSIC and (**b**) at distributed radar by using the conventional 2D MUSIC with coordinate transformation.

**Figure 12:**
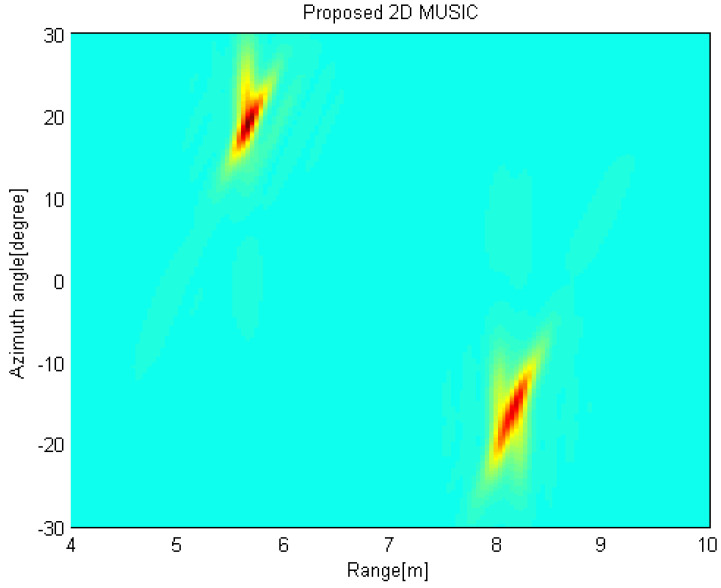
Radar image by using the proposed 2D MUSIC algorithm with coordinate transformation.

Figure 11a shows the radar image when the measured data obtained from the reference radar located at (0,0) m is exploited with the conventional 2D MUSIC algorithm. We can find that two targets are detected and the estimated targets are given as ($R_{k}$, $\theta_{k}$) = $\left\{\left(8.0\;m,-15.30^{\circ }\right),\left(5.64\;m,21.60^{\circ }\right)\right\}$. Figure 11b shows the radar image when the measured data are acquired at (2,0) m is exploited with the conventional 2D MUSIC algorithm using the coordinate transformation. Accordingly, the targets are slightly slanted. In Figure 12, the radar image is obtained by using the proposed 2D MUSIC algorithm with coordinate transformation. Radar image in Figure 12 shows that two peaks are located at $\left\{\left(8.16\;m,-15.30^{\circ }\right),\left(5.64\;m,19.0^{\circ }\right)\right\}$ with a higher resolution. That is, the target images appear more focused. Accordingly, we can estimate the targets with a high resolution without sharing the raw data of the received signals at the distributed radars.

## 6. Conclusions

In this paper, we propose the distributed 2D MUSIC algorithm with coordinate transformation to estimate the range and angle information of multiple targets with distributed FMCW MIMO radars. In the proposed scheme, by the coordinate transformation at each FMCW MIMO radar, we can proceed with the 2D MUSIC algorithm separately with respect to the reference radar’s coordinate. Accordingly, rather than reporting the raw data of the received signal to the data fusion center, the distributed radars forward their local cost function values of the 2D MUSIC for the radar image region of interest. Because the local cost function values are computed with respect to the same coordinate, their weighted sum can be computed at the data fusion center, and the range and angle information of multiple targets is jointly estimated from the aggregated cost function. In the computer simulations, when the proposed distributed 2D MUSIC algorithm is exploited, high-resolution radar images can be achieved having narrow-width peaks associated with the targets. Accordingly, it is confirmed through the Monte Carlo simulation that the proposed algorithm shows lower RMSEs than the conventional method especially at low SNR (below 10 dB), which implies that the proposed algorithm has higher immunity to the additive noise. Finally, by applying the proposed scheme to the experimentally measured data, it is verified that the range and angle parameters of multiple targets can be estimated with a high resolution without sharing the raw data of the received signals at the distributed radars.

## Figures and Tables

**Table 1:** FMCW experiment parameter setting.

Parameter	Value
Type of Signal waveform	Linear Chirped waveform
Chirp BW	1798.82 (Mhz)
Number of Chirps per frame	256
Number of Chirp Loops	120
Range resolution	0.1953 (m)
Velocity resolution	0.0472 (m/s)
Tx power	12 dBm
Sampling Rate	10,000 ksps

## Data Availability

Not applicable.
